# Synthesis of Ilmenite Nickel Titanite-Supported Carbon Nanofibers Derived from Polyvinylpyrrolidone as Photocatalyst for H_2_ Production from Ammonia Borane Photohydrolysis

**DOI:** 10.3390/polym15153262

**Published:** 2023-07-31

**Authors:** Ibrahim M. Maafa, Nasser Zouli, Ahmed Abutaleb, Ayman Yousef, Isam Y. Qudsieh, Saleh M. Matar, Abdel Samed M. Adam, M. M. El-Halwany

**Affiliations:** 1 Department of Chemical Engineering, College of Engineering, Jazan University, Jazan 45142, Saudi Arabia; nizouli@jazanu.edu.sa (N.Z.); azabutaleb@jazanu.edu.sa (A.A.); isamyq@jazanu.edu.sa (I.Y.Q.); salehmatar@yahoo.com (S.M.M.); aabaker@jazanu.edu.sa (A.S.M.A.); 2 Department of Mathematics and Physics Engineering, College of Engineering at Mataria, Helwan University, Cairo 11718, Egypt; 3 Bioprocess Development Department, Genetic Engineering and Biotechnology Research Institute (GEBRI), City of Scientific Research and Technological Applications (SRTA-City), New Borg El-Arab City, Alexandria 21934, Egypt; 4 Department of Mathematics and Physics Engineering, College of Engineering, Mansoura University, El-Mansoura 35516, Egypt; mmelhalwany@yahoo.com

**Keywords:** ilmenite, ammonia borane, polyvinylpyrrolidone, hydrogen, electrospinning, photohydrolysis

## Abstract

The present study involves the synthesis of photocatalytic composite nanofibers (NFs) comprising ilmenite nickel titanite-supported carbon nanofibers (NiTiO_3_/TiO_2_@CNFs) using an electrospinning process. The photocatalytic composite NFs obtained were utilized in hydrogen (H_2_) production from the photohydrolysis of ammonia borane (AB). The experimental findings show that the photocatalytic composite NFs with a loading of 25 mg had a good catalytic performance for H_2_ generation, producing the stoichiometric H_2_ in 11 min using 1 mmol AB under visible light at 25 °C and 1000 rpm. The increase in catalyst load to 50, 75, and 100 mg leads to a corresponding reduction in the reaction time to 7, 5, and 4 min. The findings from the kinetics investigations suggest that the rate of the photohydrolysis reaction is directly proportional to the amount of catalyst in the reaction system, adhering to a first-order reaction rate. Furthermore, it was observed that the reaction rate remains unaffected by the concentration of AB, thereby suggesting a reaction of zero order. Increasing the reaction temperature results in a decrease in the duration of the photohydrolysis reaction. Furthermore, an estimated activation energy value of 35.19 kJ mol^−1^ was obtained. The composite nanofibers demonstrated remarkable and consistent effectiveness throughout five consecutive cycles. The results suggest that composite NFs possess the capacity to function as a feasible substitute for costly catalysts in the process of H_2_ generation from AB.

## 1. Introduction

Hydrogen (H_2_) energy is an environment-friendly source of energy that is gaining interest across the globe because of its potential to alleviate the energy crisis, facilitate the transformation of the energy sector, and mitigate the effects of climate change and pollution [1,2,3]. The use of a H_2_ economy in our day-to-day lives is still meeting a great number of challenges, the foremost of which is the storage of H_2_ [4,5]. A possibility is to store H_2_ in the form of solid-state H_2_ storage, which relies on either physical or chemical interactions between H_2_ and storage materials [6,7]. Among these, amine-borane stands out as an effective hydrogen storage material because it satisfies a number of requirements set by the United States Department of Energy (DOE): (a) possessing a significant amount of hydrogen, (b) stability, and (c) eco-friendliness [8,9]. Ammonia borane (NH_3_BH_3_) is the most basic form of the amine-borane compounds that is being studied for its potential use in solid hydrogen storage material due to its high (19.6 wt.%) hydrogen content [10,11]. Hydrogen produced by the photocatalytic hydrolysis of AB with the help of light has attracted a lot of attention because it is a promising clean, efficient, and infinite energy source [11,12]. Ayman et al. published the first proof of AB photohydrolysis in 2012 [13]. Since then, several studies have been conducted to determine the efficacy of various photocatalytic substances in generating hydrogen from AB [14,15,16]. The photohydrolysis method can produce gravimetric hydrogen capacity in AB, which is comparable to what happens during normal hydrolysis; however, radiation from light could be able to restrict the generation of ammonia [17]. Various metal- and non-metal-supported TiO_2_ have been used as efficient photocatalysts for the production of H_2_ from the photohydrolysis of AB [14,17,18,19,20,21,22]. Over the last several years, ilmenite has been developed as an effective visible-light-driven photocatalyst [23,24,25,26]. Because of its adequate bandgap and capacity to absorb solar radiation, nickel titanate (NiTiO_3_, Eg~2.18 eV) has been identified as a suitable photocatalytic material for various reactions [27]. Huang et al. [28] produced NiTiO_3_/TiO_2_ nanotubes and used them to produce H_2_ by photosplitting. The fabricated NiTiO_3_/TiO_2_ showed promising photocatalytic performance with H_2_ generation rates of 45 and 680 mol g^−1^ h^−1^ for TiO_2_ and NiTiO_3_/TiO_2_, respectively. Carbon nanofibers (CNFs) have been demonstrated to possess excellent electrical conductivity, which means that they may readily gather and transfer photo-induced charges during the photocatalytic process [29]. In this investigation, we report for the first time on the production of NiTiO_3_/TiO_2_@CNFs and their photocatalytic activity towards AB photohydrolysis. The catalyst is synthesized employing an electrospinning approach. The produced NFs exhibited excellent photocatalytic activity for H_2_ generation via AB photohydrolysis.

## 2. Experimental

### 2.1. Preparation of Composite NFs 

The sol–gel method was used to manufacture TiO_2_-decorated CNFs. This was accomplished by combining 20 g of 12% solution of polyvinylpyrrolidone (PVP, 99.5%, Sigma Aldrich, USA) with 3 mL titanium isopropoxide (TIIP, 97%, Sigma Aldrich, USA). The PVP solution was prepared following the procedures described in other studies [30,31,32,33,34,35]. After stirring the mixture for an adequate duration of time, a translucent yellow gel is formed. In the laboratory-scale electrospinning apparatus, the formed sol–gel is introduced into the plastic syringe. The positive electrode is attached to the metallic tip of the plastic syringe, while the negative electrode is attached to a rotating metallic cylinder enclosed within wax foil. Throughout the spinning process, the potential and space between the positive and negative electrodes were kept as 20 kV and 15 cm, respectively. After the NF mats had formed on the foil, they were peeled off and dried in a vacuum drier overnight at 60 °C. The material was then calcined under vacuum in an Ar atmosphere at 950 °C for 5 h. Following the same protocol as before, nickel acetate tetrahydrate (NiAc, 98.5%, Sigma Aldrich, USA) was added and stirred. The same electrospinning and calcination methods were used once a translucent green solution was obtained.

### 2.2. Characterization

The synthesized composite was characterized using scanning electron microscopy, X-ray diffraction, and transmission electron microscopy. The details of these techniques have been discussed in our previous work [35].

### 2.3. Photohydrolysis of AB

The photocatalytic performance of the prepared NFs for H_2_ production employing the photohydrolysis of AB was investigated. An LED (λ = 365 nm) light served as the source of illumination. A three-neck flask was used as the reactor. The water-displacement method was used to evaluate the volume of H_2_ produced. After adding 1 mmol of ammonia borane complex (AB, 97.0%) and 25 mg of catalyst to the reaction reactor, the charge was subjected to visible light to determine the produced H_2_. In order to eliminate ammonia, the formed H_2_ was passed through a 0.001 M HCl solution. There are several factors that have been studied, such as the concentration of AB, amount of catalyst, and reaction temperature.

## 3. Results and Discussion

**Figure 1:**
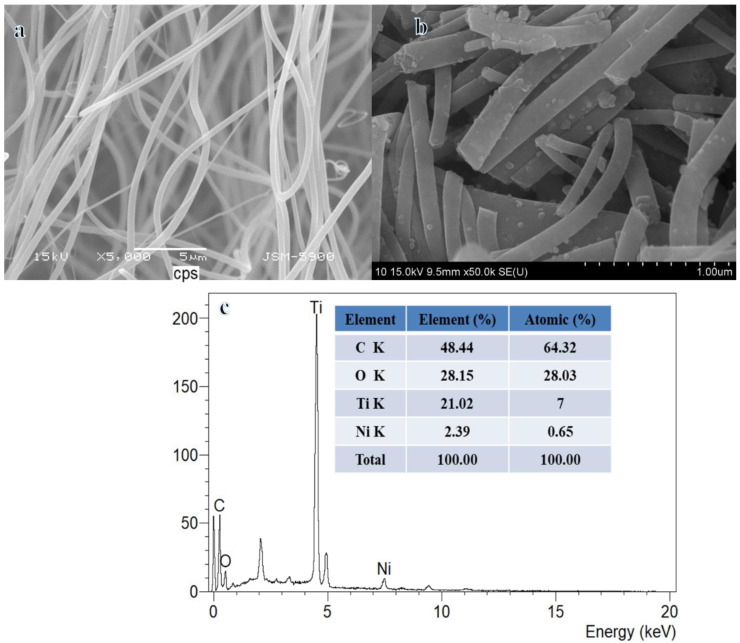
(**a**) SEM image of TIIP-PVP, (**b**) FESEM image, and (**c**) EDX analysis of composite NFs obtained after sintering at 950 °C in Ar.

Electrospun nanofibers (NFs) composed of TIIP-PVP, as shown in Figure 1a, are smooth and bead-free following sintering at 950 °C for 5 h in a vacuum in an Ar atmosphere. The FESEM image displayed in Figure 1b was acquired after the calcination of electrospun NFs composed of NiAc, TIIP, and PVP at 950 °C for five hours in a vacuum containing argon. As can be seen in Figure 1a, the NFs maintained their structure with the growth of very small NPs on the surface of the NFs. According to the results of the EDX study (Figure 1c), the NFs are mostly composed of nickel (Ni), titanium (Ti), oxygen (O), and carbon (C), and no additional elements have been found. FESEM-EDX images (Figure 1b,c) showed the formation of NPs on the surface of NFs. The EDX analysis demonstrated the presence of Ti, O, C, and Ni elements in the spectra. It is possible that the presence of carbon is making the photocatalytic process more efficient overall by doing the following: increasing the adsorption of AB molecules, which in turn increases the rate at which they are hydrolyzed by the photo-inducement of e− and h+ separation.

**Figure 2:**
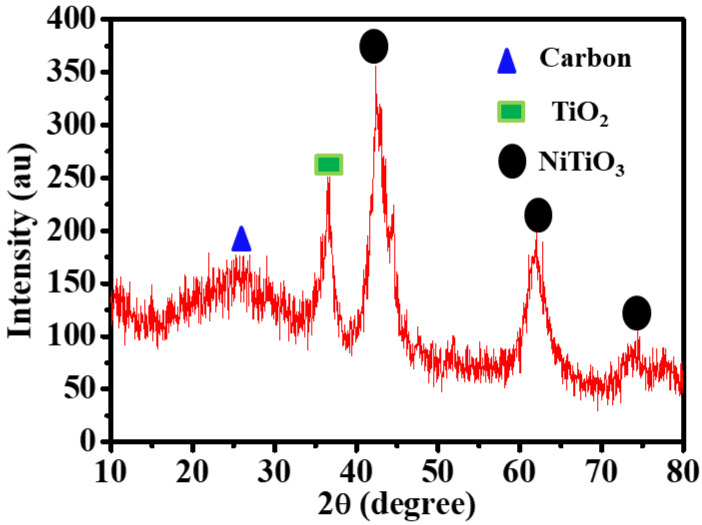
XRD analysis of composite NFs after sintering at 950 °C in Ar.

The XRD data of the composite that was produced after the calcination procedure are shown in Figure 2. One peak of the TiO_2_ phases, the rutile phase (JCPDS #00-004- 0551), was found to develop at a 2θ of 36.65°, matching the (101) crystal plane. Furthermore, a hexagonal NiTiO_3_ phase of ilmenite (JCPDS #12035-39-1) occurred at 2θ of 42.32°, 62.09°, and 74.47°, corresponding to the (021), (214), and (217) crystal orientations, respectively. The NPs’ sizes were determined to be 8.43 nm using Scherrer’s equation. Carbon-like graphite, which develops as a result of a partial degradation of carbon during the calcination process, has an outside peak at a 2θ that agrees with (002). The XRD of TiO_2_@CNFs has been shown in our previous work [36].

### 3.1. Photodehydrogenation of AB

**Figure 3:**
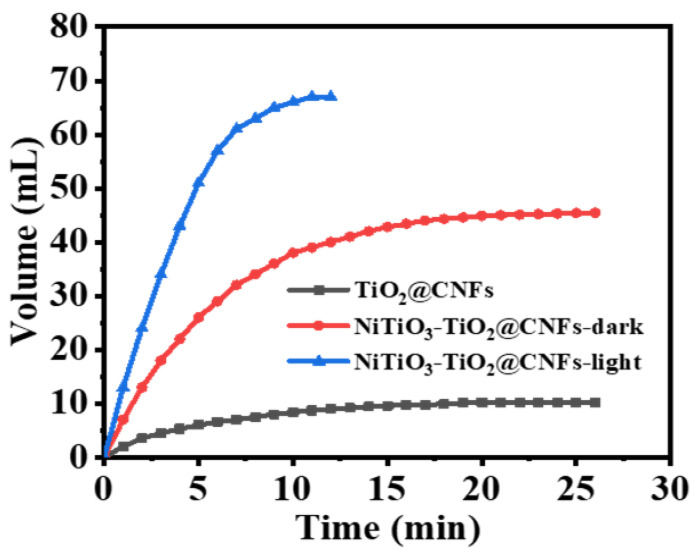
Volume of H_2_ production from AB solution vs. time in the existence of photocatalysts under light irradiation (25 mg of photocatalyst, 25 °C, 1 mmol AB, and 1000 rpm).

It is interesting to mention that CNFs have been used as a supporting matrix in different reactions, but they did not exhibit any catalytic activity towards the dehydrogenation of AB, whereas a Ni-based catalyst demonstrated superior performance in both the catalytic and photocatalytic processes involved in H_2_ production from AB [13,37,38]. This study evaluated the photodehydrogenation efficiency of pristine TiO_2_ and composite NFs in the process of releasing hydrogen from the hydrolysis of AB (1 mmol AB) under visible illumination (λ = 365 nm) at a temperature of 25 °C and 1000 rpm (Figure 3). The total volume of H_2_ produced with regard to pristine TiO_2_ and composite NFs have been estimated to be 9 and 67 mL within a 12 min period, respectively. The activity of composite nanofibers was found to be significantly higher in comparison to pristine TiO_2_. 

The observed phenomenon may be attributed to the rapid separation of electron–hole pairs in composite nanofibers, which causes an increased number of ions in the solution, thereby enhancing hydrogen production via photodehydrogenation of AB in comparison to pristine TiO_2_. The free radicals created in the solution upon exposure to sunlight could be the reason for this observation; these radicals assault the AB molecules in order to release hydrogen. The finding that the generation of H_2_ was notably augmented when subjected to visible illumination in contrast to dark conditions is a topic of interest. In the absence of a catalyst, the stability of AB in water was evidenced by the absence of H_2_ detection even under visible illumination.

### 3.2. Influence of Composite NF Loading

**Figure 4:**
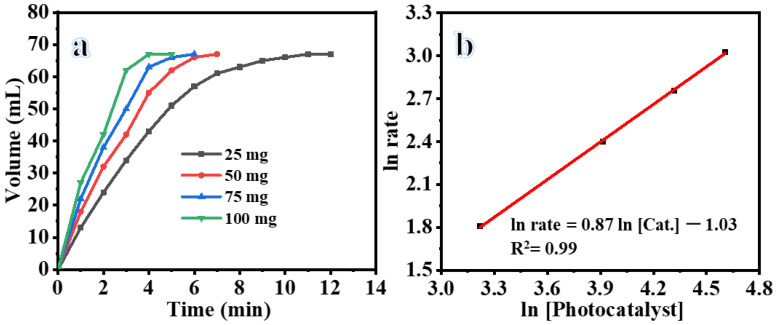
(**a**) Influence of photocotalyst on H_2_ production, and (**b**) H_2_ production rate vs. amount of photocatalyst on logarithmic scale (25 °C, 1 mmol AB, and 1000 rpm).

Figure 4a depicts the relationship between the equivalent produced H_2_ and the duration of exposure to light at varying loads of composite NFs (25, 50, 75, and 100 mg). The results indicate that a rise in the load of composite NFs leads to a corresponding rise in the rate of H_2_ generation. Furthermore, the four doses demonstrated activities with the following Turn Over Frequency (TOF) values: 12.84, 22.02, 25.69, and 38.53 mol_(H2)_ mol_(cat)_^−1^ min^−1^ for 25, 50, 75, and 100 mg of composite NFs, respectively. This phenomenon can be attributed to the enhanced surface area of the composite NFs, which enhances the photodehydrogenation of AB. 

Figure 4b illustrates the natural logarithm of the reaction rate plotted against the natural logarithm of the composite NF load. The calculated slope of the optimal regression line is 0.87, indicating that the photodehydrogenation of H_2_ conforms to the principles of pseudo-first-order kinetics with respect to the catalyst loading.

### 3.3. Influence of AB Concentration 

**Figure 5:**
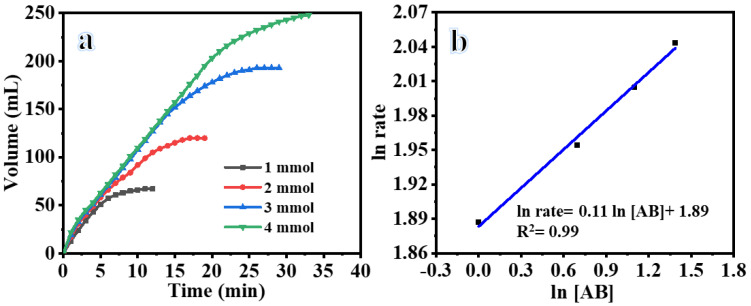
(**a**) Influence of AB concentration on H_2_ production, and (**b**) H_2_ production rate vs. AB concentration on logarithmic scale (25 mg of photocatalyst, 25 °C, and 1000 rpm).

The impact of varying initial AB concentrations (1, 2, 3, and 4 mmol) on the generation of H_2_ through AB photodehydrogenation under visible illumination while utilizing composite NFs was investigated (Figure 5a). As illustrated in the graph, the concentration of AB did not have a significant impact on the rate of H_2_ production. Specifically, the initial rate of H_2_ generation remained relatively constant despite an increase in AB concentration. The utilization of varying AB concentrations in the photogeneration of H_2_ can be attributed to a pseudo-zero-order reaction, as depicted in Figure 5b. The calculated slope of the regression line was 0.11, indicating that the hydrogen production rate adheres to pseudo-zero-order kinetics related to AB.

### 3.4. Influence of Reaction Temperature

**Figure 6:**
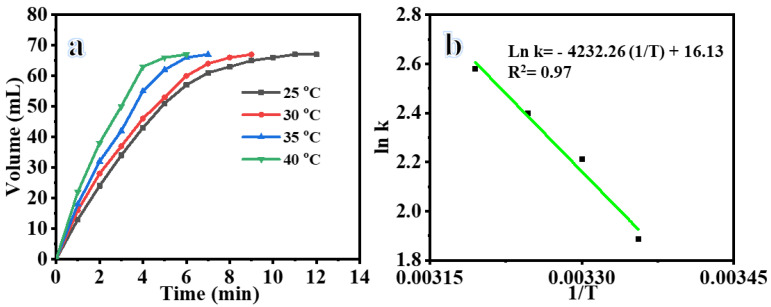
(**a**) Influence of reaction temperature on H_2_ production, and (**b**) ln (*k*, rate constant) vs. temperature inverse (25 mg of photocatalyst, 1 mmol AB, and 1000 rpm).

Figure 6a plots the H_2_ production rate vs time in the presence of composite NFs at different temperatures. The figure illustrates that the photodehydrogenation of AB was observed to rise as the reaction temperature was elevated from 25 to 40 °C. This can be attributed to the enhanced mobility of charge carriers and the interfacial transfer of charges at higher temperatures. With the rise in reaction temperature, there is a corresponding rise in the mobility of photoelectron–hole pairs. This results in a more rapid combination of electrons with adsorbed oxygen and a quicker generation of OH radicals in conjunction with -OH [39,40,41]. Consequently, the AB photodehydrogenation process is enhanced. A linear correlation is observed between the values of K and 1/T, as depicted in Figure 6b. estimated activation energy (E_a_) is 35.19 KJ mol^−1^. 

### 3.5. Catalyst Recyclability Data 

**Figure 7:**
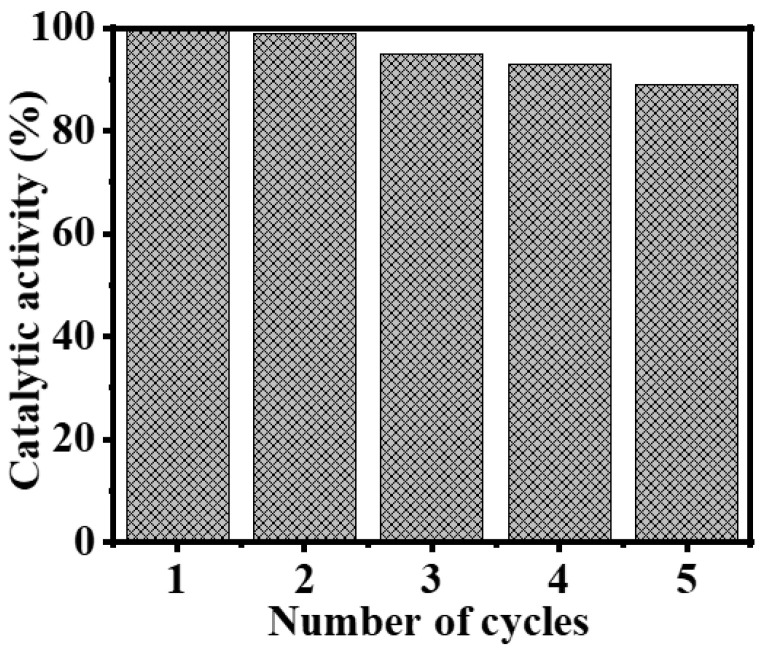
Catalyst recyclability data of composite NFs (25 mg of photocatalyst, 25 °C, 1 mmol AB, and 1000 rpm).

In order to investigate the extended stability and recyclability of the fabricated composite NFs, a series of photocatalytic experiments were conducted using the composite NFs over the course of five cycles (Figure 7). As depicted in Figure 7, a notable photocatalytic response was observed, accompanied by a minimal reduction in the catalytic performance. This observation shows that the newly developed photocatalytic composite NFs exhibit longevity in the catalytic reaction, and the photodegradation activity of the catalyst showed a little decrease (~10%) after five runs. The data show that the catalyst is stable and recyclable. 

### 3.6. Comparison of Our Results with the Literature

The catalytic activity of the current composite NF catalyst for H_2_ production was compared with others reported in the literature, as shown in Table 1. The results obtained from the photohydrolysis of AB in the presence of catalyst NiTiO_3_/TiO_2_-decorated CNFs for H_2_ production were compared with the catalysts reported in the literature with titanate as an active ingredient. Chandra et al. demonstrated the photocatalytic activity of Rh/g-Al_2_O_3_ in the photohydrolysis of AB to produce H_2_ at room temperature [42]. Their study determined the activation energy to be 21 kJ mol^−1^. Zhu et al. synthesized Cu–ZrO_2_ xerogel photocatalysts via a facile two-step approach involving an epoxide-driven sol–gel method followed by chemical reduction [43]. The 15% Cu–ZrO_2_ showed the best catalytic activity with a maximum H_2_ production rate of 0.384 mol_(H2)_ mol_(cat)_^−1^ min^−1^. The kinetics study revealed that the photohydrolysis of AB followed first-order kinetics when 15% Cu–ZrO_2_ xerogel was used, with an activation energy of 22.34 kJ mol^−1^. Their catalyst was stable and recyclable even after five cycles of the experiment. Chen et al. synthesized a SiO_2_-encompassed Co@N-doped porous carbon catalyst. This novel recyclable catalyst was further employed for the calcination of zeolitic imidazolate framework-67@SiO_2_ microtubes at high temperatures in a N_2_ atmosphere [44]. The catalyst obtained from the calcination at 800 °C exhibited a high H_2_ generation rate of 8.4 mol_(H2)_ mol_(cat)_^−1^ min^−1^ with an activation energy of 36.1 kJ mol^−1^. Gao et al. synthesized monodisperse Ni nanoparticles via a facile technique and then anchored them on graphitic carbon nitride (g-C_3_N_4_) nanosheets through a self-assembly process [37]. They determined an optimum AB photohydrolysis rate corresponding to a 3.2 nm size of Ni NPs with a TOF of 18.7 mol_(hydrogen)_ mol_(catalyst)_^−1^ min^−1^ and an activation energy of 36 kJ mol^−1^. Wang et al. employed TiO_2_(B) nanotubes (NTs) that supported metal Cu/Ni nanoparticles for the catalytic hydrolysis of ammonia borane under visible light [45]. They synthesized the TiO_2_ NTs via a hydrothermal technique and then loaded them with Cu/Ni metal nanoparticles via the impregnation–reduction method. The H_2_ generation rate of 15.90 mol_(H2)_ mol_(cat)_^−1^ min^−1^ was obtained using Cu_0.64_Ni_0.36_-TiO_2_ NTs with an activation energy of 36.14 kJ mol^−1^. In another study, Wang et al. employed TiO_2_ with monoclinic-phase (TiO_2_(B)) and anatase-phase composite (TiO_2_(B)/anatase) loaded with 3.66 wt.% CuNi alloy nanoparticles (NPs) as a photocatalyst for hydrogen generation from ammonia borane [19]. The as-synthesized Cu_0.36_Ni_0.64_-T700 catalysts exhibited the highest H_2_ generation rate of 21.87 mol_(H2)_ mol_(cat)_^−1^ min^−1^ with an activation energy of 27.40 kJ mol^−1^. Cheng et al. employed MoO_3−_*_x_* nanosheets to enhance the hydrogen production rate from ammonia borane under visible light [46]. They obtained a TOF of 5.74 mol_(H2)_ mol_(cat)_^−1^ min^−1^. Lu et al. immobilized Cu–Ni nanoparticles (NPs) in MCM-41 employing a liquid impregnation–reduction technique [47]. The produced composite Cu_0.2_Ni_0.8_/MCM-41 was utilized for the generation of H_2_, and it performed well, yielding a TOF of 10.7 mol H_2_ (mol cat)^−1^ min^−1^ with an activation energy of 38 kJ mol^−1^.

### 3.7. Photodehydrogenation Mechanism

It has been hypothesized that the composite NFs would undergo the photohydrolysis process of AB photodehydrogenation. The process of separating electron (e^−^)–hole (h^+^) pairs produced by visible illumination in the composite NFs can be understood by evaluating the conduction band (CB) and valence band (VB) potentials of the constituent materials. The energies in controversy were computed utilizing the subsequent empirical formulas [48]:

E_CB_ = χ − E_e_ − 0.5E_g_ (1)

E_VB_ = E_CB_ + E_g_ (2)

**Figure 8:**
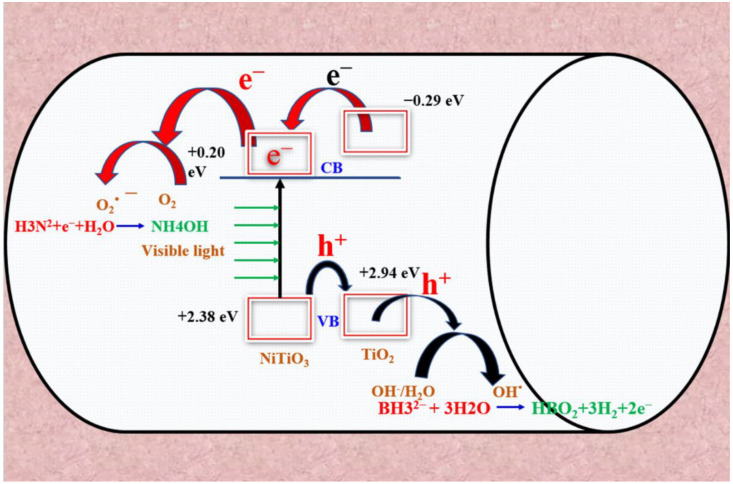
Schematic representation of the photodehydrogenation of AB.

Equation (1) represents the relationship between the conduction band energy (E_CB_), the electron energy (E_e_), and the bandgap energy (E_g_) in the context of the E_CB_. Equation (2) represents the relationship between the valence band energy (E_VB_) and E_CB_, taking into account the bandgap energy. Furthermore, the energy of liberated electrons in relation to the Normal Hydrogen Electrode (NHE) is denoted as Ee and measures 4.5 electron volts [48]. The bandgap energy of the semiconductor is denoted by “Eg”. The symbol χ is utilized to represent the electronegativity of the semiconductor. According to a study, the potentials of CB and VB of ilmenite nickel titanite in comparison to SHE were determined to be +0.20 eV and +2.38 eV, respectively. Ilmenite nickel titanite exhibits a lower bandgap of 2.62 eV in comparison to pristine TiO_2_, which has a bandgap of 3.2 eV. As a result, the VB position of ilmenite nickel titanite is higher at +2.38 eV in contrast to TiO_2_, which is at +2.94 eV. This higher VB position of ilmenite nickel titanite facilitates the transfer of h^+^ from its VB to the VB of TiO_2_. Upon photoexcitation, the photogenerated h^+^ undergoes a reaction with H_2_O/OH^−^, resulting in the production of OH radicals [39]. It is noteworthy that the CB of ilmenite nickel titanite is situated at a lower energy level than that of TiO_2_ (−0.29 eV). Upon exposure to light, the VB e^−^ in ilmenite nickel titanite undergo excitation and transition to the CB, leading to the partial vacancy of the VB. Consequently, e^−^ from the CB of TiO_2_ are transferred to the CB of ilmenite nickel titanite. Its involvement in the photodehydrogenation process implies its potential as a proficient sensitizer that can effectively harness visible illumination. The mobility of photogenerated e^−^ in ilmenite nickel titanite is observed to be directed towards the surface of the CNFs, indicating a high degree of separation efficiency between photogenerated e and h^+^ with low rates of recombination [40]. The O_2_ molecules undergo a reaction with the photogenerated electron located in the conduction band of ilmenite nickel titanite, resulting in the production of •O_2_^−^ without any recombination with the holes that are present on the surface of TiO_2_ [37]. The resulting oxide anions (•O_2_^−^) undergo a reaction with hydrogen ions, leading to the formation of hydroperoxyl radicals (HOO•), which subsequently induce modifications to the AB molecule present in the solution. According to a study, it was found that the composite NFs exhibited a greater level of photocatalytic activity compared to that of the pristine TiO_2_. The mechanism of photodehydrogenation of AB utilizing composite NFs can be demonstrated as presented in Figure 8 [13,15,37,41,49].

## 4. Conclusions

In this study, photocatalytic composite NFs consisting of NiTiO_3/_TiO_2_@CNFS were synthesized through the electrospinning technique and used for H_2_ generation from AB. The use of photocatalytic composite NFs in the photohydrolysis process resulted in a significant increase in the catalytic efficiency and a substantial production of H_2_ compared to pristine TiO_2_. Furthermore, the photocatalytic composite NFs exhibited remarkable photocatalytic performance, as indicated by the estimated low activation energy (35.19 kJ mol^−1^). The experimental findings show that the photocatalytic composite NFs with a loading of 25 mg had a good catalytic performance for H_2_ generation, producing stoichiometric H_2_ in 11 min using 1 mmol AB under visible light at 25 °C and 1000 rpm. An increase in catalyst loading to 50, 75, and 100 mg led to a corresponding reduction in the reaction time to 7, 5, and 4 min, respectively. The findings from the kinetics investigations suggest that the rate of the photohydrolysis reaction is directly proportional to the amount of catalyst in the reaction system, adhering to a first-order reaction rate. Furthermore, it was observed that the reaction rate remains unaffected by the concentration of AB, thereby suggesting a reaction of zero order. Increasing the reaction temperature results in a decrease in the duration of the photohydrolysis reaction. The composite nanofibers demonstrated remarkable and consistent effectiveness throughout five consecutive cycles. The results suggest that composite NFs possess the capacity to function as a feasible substitute for costly catalysts in the process of H_2_ generation from AB. The photocatalytic composite NFs demonstrated a consistent and enduring production of H_2_ throughout reusability and stability assessments for five cycles. This suggests that they have the potential to serve as viable photocatalysts for practical uses.

## Figures and Tables

**Table 1:** Performance comparison of our catalyst with others reported in the literature for H_2_ production from AB.

Catalyst	TOF, mol_(H2)_ mol_(cat)_^−1^ min^−1^	E_a_, kJ mol^−1^	Ref.
Rh/g-Al_2_O_3_	-	21	[42]
CuZrO_2_	0.384	22.34	[43]
Co@CeN@SiO_2_	8.4	36.1	[44]
Ni/g-C_3_N_4_	18.7	36	[37]
Cu_0.64_Ni_0.36_-TiO_2_(B) NTs	15.9	36.14	[45]
Cu_0.36_Ni_0.64_-T700	21.87	27.40	[19]
MoO_3_-x	5.74	--	[46]
Cu_0.2_Ni_0.8_/MCM-41	10.7	38	[47]
NiTiO_3_/TiO_2_@CNFs	22.02	35.19	This Work

## Data Availability

Not applicable.
